# Care Coordination Satisfaction Survey for Families of Children and Youth with Special Healthcare Needs

**DOI:** 10.1007/s10995-025-04153-z

**Published:** 2025-09-05

**Authors:** Molly Hofmann, Patricia C. Perez, Ruann Barack

**Affiliations:** 1 Division of Specialized Care for Children, University of Illinois at Chicago, 3135 Old Jacksonville Rd, Springfield, IL 62704 USA; 2 Division of Specialized Care for Children, University of Illinois at Chicago, 4700 N Sterling Ave, Peoria, IL 61615 USA

**Keywords:** CYSHCN, Care coordination, Family partnership, Family survey

## Abstract

**Introduction:**

Care coordination can be an essential source of support to families of children with special health care needs and should ideally lead to improvements in the organization of care.

**Purpose:**

This publication aims to share the experience of one statewide Title V organization’s work to develop and validate a care coordination satisfaction survey.

**Description:**

UIC-DSCC engaged with the Family Advisory Council and a Family Survey Committee to revise existing surveys measuring different care coordination domains. The overall consensus was to apply a 13-item annual care coordination satisfaction survey. The survey was administered to families between January and April 2023. Psychometric reviews included exploratory Factor Analysis for the underlying structure of the items and Cronbach α for reliability. A top-box approach was used to represent item proportion.

**Assessment:**

899 families completed the 13-item measure. The Exploratory Factor Analysis determined a 2-factor solution: (1) Care Coordination Satisfaction and (2) Engagement and Impact on Quality of Life—none of the items required removal. Factor 1’s top-box results show families’ positive experience with care coordination, with 6.37 of the seven questions being answered with the most favorable answer. Similarly, in factor 2, 5.28 of the six questions were responded to with the top or most favorable answer.

**Conclusion:**

Family partnership is a crucial part of the care coordination process, and UIC-DSCC has learned that this partnership is also critical when evaluating family satisfaction with care coordination services. The 13-item care coordination survey demonstrated adequacy and can assist with quality improvement in care coordination programs.

## Significance

We developed and validated a care coordination satisfaction survey for families of children and youth with special healthcare needs enrolled in a statewide Title V Care Coordination program. The survey instrument can guide quality improvement efforts by other Title V Care Coordination agencies operating outside a clinical setting and targeting a similar population.

## Introduction

Care coordination has become an increasingly common method of providing support for children with special health care needs (CYSHCN) and their caregivers. Ideally, care coordination is a dynamic process that helps to support the children and their family’s full range of social, behavioral, environmental, and health care related needs (NASHP, 2020). Care coordination can be made available through various opportunities, including as part of the child’s medical home, through health insurance, from schools, through state Title V programs, or through various community-based organizations. Family partnership is a crucial part of the care coordination process and is important to be included when measuring outcomes related to care coordination (NASHP, 2020).

Nearly 20% of children in the United States have a special healthcare need, with less than 15% of this population’s caregivers reporting that their child receives care in a well-functioning system (McLellan et al., 2022). The recently released Maternal Child Health Bureau Blueprint for Change suggests that measurements looking at family quality of life, caregiver well-being, and partnership are important when evaluating the systems of care supporting CYSHCN and their families and are needed in consideration for how to drive improvements in care moving forward (Coleman et al., 2022).

The University of Illinois Chicago Division of Specialized Care for Children (UIC-DSCC) is the state of Illinois’ Title V Program serving CYSHCN. UIC-DSCC currently operates three different care coordination programs serving various populations of CYSHCN across Illinois (Table 1). Care coordination is defined by UIC-DSCC as a person and family-centered, strength-based, assessment-driven approach to empowering families to achieve their goals that ultimately lead to positive health outcomes, improved quality of life, and overall family satisfaction. As part of ongoing quality improvement, UIC-DSCC uses a care coordination family satisfaction survey to seek feedback from program participants and their families. The survey is intended to provide UIC-DSCC with feedback from families related to their care coordination services, which includes questions focused on the family’s perception of partnership in the person-centered planning process, communication, overall satisfaction with DSCC’s care coordination services, and how UIC-DSCC care coordination impacts their family’s overall quality of life. A review of the literature demonstrates that little has been published about the development, validation, and implementation of a family satisfaction survey to assess the effectiveness of care coordination services by a Title V care coordination agency outside of clinical settings such as a hospital or outpatient clinic. This publication describes one care coordination program, UIC-DSCC’s, efforts to develop and validate a family survey to use in the evaluation of satisfaction with care coordination services within a Title V organization that serves CYSHCN families outside clinics or hospital sites throughout the state of Illinois. It also seeks to share lessons learned about family engagement and partnership roles in the survey process.

**Table 1:** UIC-DSCC care coordination programs

Programs	Population served
Home care program	UIC-DSCC operates this program on behalf of the state’s Medicaid program. This program provides care coordination for individuals who qualify for in-home, shiftbased nursing through Medicaid.
Core program	Serves children ages 0-21 years who have a chronic condition that falls into one of the following categories of eligibility: blood disorder, inborn errors of metabolism, cardiovascular impairment, neurologic impairment, craniofacial and external body impairments, eye impairments, gastrointestinal impairments, hearing impairments, pulmonary impairment, urogenital impairment. Participants in the Core Program may not be enrolled in a Medicaid Managed Care Program.
Connect care program	Eligibility for this program is the same as the Core Program, except the child must be enrolled in a Medicaid Managed Care Program that UIC-DSCC is contracted with.

## Methods

### Survey Development

In January 2018, a Family Survey Committee (FSC) was formed to develop a series of family surveys that could be utilized across all UIC-DSCC programs. The committee included 10 employees who were located in different areas of the state and in various positions, from care coordination to quality improvement. It also included a parent of a DSCC participant. The committee’s goal was to create surveys focused on different aspects of care, such as education and transition, and that provided feedback on care coordination services at different times during their program enrollment, such as the beginning and upon disenrollment. The committee developed the following family surveys: initial, annual, transition, education, exit, and home visit. Surveys were formatted with a blend of Likert scale items, along with two open-ended questions about what was working and how we could improve. The survey response options were 5-point Likert scales that were 5 (strongly agree), 4 (somewhat agree), 3 (neither agree nor disagree), 2 (somewhat disagree), and 1 (strongly disagree). The committee worked to ensure there was consistency in the questions across surveys, such as quality of life and satisfaction with care coordination.

The initial, annual, and exit surveys were the first to be implemented in 2019. Each survey had a specific timeframe in which the survey was to be sent to the family. For instance, the initial survey was sent to the family within 60 days of enrollment, the exit survey was sent within 30 days of discontinuation, and the annual survey was sent at 1 year, 3 years, 5 years, and then every 5 years based upon enrollment with DSCC. In 2020, the transition and education surveys were implemented. Each of these also had designated timelines to be distributed to the families.

In 2022, UIC-DSCC observed a need to do something different to improve the engagement and rate of response to family surveys. Through this improvement phase, UIC-DSCC partnered with the Family Advisory Council (FAC), along with the internal Family Survey Committee. The UIC-DSCC FAC was parent-chaired and had 17 committee members at that time. Family members on the council were all English-speaking and were primarily female (2 were male). The FAC members represented different regions of the state, including urban and rural communities, and had children enrolled in one or more of DSCC’s care coordination programs with ranging types of special healthcare needs. The UIC-DSCC team shared with the FAC about the need to hear more from families about care coordination services, in order to continue to improve these services. Council members shared feedback about their experience receiving satisfaction surveys from UIC-DSCC and from other places where their children receive care. Common themes in the feedback from FAC members included the desire to be provided information about specific ways the survey informed change, feelings of survey fatigue from the number of surveys families receive from providers involved in their child’s care, a desire to have more electronic access to complete the survey, a need for more reminders to complete the survey, and the FAC provided feedback on ways to improve the readability of the survey.

It was decided that the feedback and improvements would focus primarily on the annual survey. Several improvements were made. First, it was decided to make the annual survey once a year for all programs and families instead of being based on enrollment timelines. This allowed the ability to focus on strengthening communication with families through social media and from UIC-DSCC care coordination teams. Additionally, the letter that went with the survey was enhanced to include examples of how UIC-DSCC had improved care coordination in response to family feedback. We also implemented friendly reminders to those who had yet to reply. A Family Survey section was created on the UIC-DSCC website dedicated to informing families and external partners about the care coordination surveys. As a result of feedback about survey fatigue, the education survey was eliminated, and a few education-related questions were instead added to the annual survey. Prior to finalizing the updated survey, the internal Family Survey Committee provided one last review to ensure readability and clarity of the questions. Upon completion of this review, the survey included 13 questions assessing care coordination satisfaction. The questions were evaluated using the aforementioned 5-point Likert scale and two open-ended questions for additional comments. Interested families who completed a survey could be entered into a drawing to win one of five fifty-dollar Visa gift cards.

The UIC-DSCC family survey process is part of ongoing organizational quality improvement and is intended to inform improvements in the care coordination services provided by UIC-DSCC. Therefore, it does not require Institutional Review Board approval. This publication is intended to share the experience of this one organization.

### Analysis

Statistical analyses were conducted using Statistical Package for Social Sciences (SPSS) version 29.0. Chi-square test for categorical variables and the ANOVA test on continuous variables (*p* <.005) were used to examine demographic differences through programs (Connect Care, Core, and Home Care). Psychometric analysis included Exploratory Factor Analysis (EFA) with Promax rotation to determine the underlying structure of the 13 items. Factor loadings (> 0.40) were used for mapping items to a construct. We inspected Eigenvalues (> 1) and scree plot for the appropriate number of factors (Appendix Fig. 1). Cronbach’s α for reliability estimates were also examined. No items were removed through this process. A top box approach representing the proportion of items was applied for each factor (McDaniel et al., 2023). Scores from 0 to 100 were considered, where high scores indicated better experience with care coordination services. Correlations between factor scores and demographics were also studied. The 2 qualitative open-ended questions included in the survey were not a part of this analysis.

## Results

Survey participation lasted from January to April 2023 reaching 3,765 unique families. Surveys were distributed in English and Spanish. The Spanish translation was completed by a certified language interpreter agency contracted with UIC. Subsequent reminders were sent to anyone who had not yet responded (February, March, and April). Most surveys, including reminders, were delivered via email (*N* = 3368, 89%) compared to (*N* = 872, 23%) mail. In total, 899 UIC-DSCC families completed the survey, with an overall response rate of 24%; the response rate to emails was 23% (*N* = 773), and 14.4% with mail (*N* = 126). Demographic characteristics are provided in Table 2. The EFA determined a 2-factor solution (Appendix Table 6) with 7 items for Care Coordination Satisfaction (CCS), Cronbach α of 0.95, and 6 items loaded to Family Engagement and Partnership (FEP), Cronbach α of 0.94 (Table 3).

**Table 2:** Demographic characteristics of UIC- DSCC participants

	Overall sample	Connect care	Core	Home care	*p*-value
	*N*= 899	*N*= 271	*N* = 348	*N*= 280	
**Age**,** y**,** Mean** (SD)	11.6 (6)	12.5 (4.6)	11.18 (5.5)	11.39 (7.5)	0.012
**Gender**, N(%)					0.96
Female	399 (44.4)	120 (44.3)	153 (44)	126 (45)	
Male	500 (55.6)	151 (55.7)	195 (56)	154 (55)	
**Region**^a^, N(%)					0.009
Urban	661 (73.5)	196 (72.3)	241 (69.3)	224 (80)	
Rural	238 (26.5)	75 (27.7)	107 (30.7)	56 (20)	
**Ethnicity**, N(%)					0.001
White	401 (44.6)	91 (33.6)	188 (54)	122 (43.6)	
Black	118 (13.1)	39 (14.4)	16 (4.6)	63 (22.5)	
Hispanic	189 (21)	91 (33.6)	34 (9.8)	64 (22.9)	
Other ^b^	191 (21.2)	50 (18.5)	110 (31.6)	31 (11.1)	
**Language**, N(%)					0.001
English	804 (89.4)	228 (84.1)	331 (95.1)	245 (87.5)	
Other	95 (10.6)	43 (15.9)	17 (4.9)	35 (12.5)	
**Insurance**, N (%)					0.001
Private	432 (48.1)	12 (4.4)	324 (93.1)	96 (34.3)	
Public	467 (51.9)	259 (95.6)	24 (6.9)	184 (65.7)	
Endocrine, nutritional, metabolic, N (%)	77 (8.6)	16 (5.9)	20 (5.7)	41 (14.6)	0.001
Mental, Behavioral, Neurodevelopmental, N(%)	64 (7.1)	23 (8.5)	18 (5.2)	23 (8.2)	0.19
Genitourinary, N (%)	79 (8.8)	27 (10)	25 (7.2)	27 (9.6)	0.39
Nervous System, N (%)	401 (44.6)	124 (45.8)	124 (35.6)	153 (54.6)	0.001
Ear, Eye, Mastoid Process, N(%)	355 (37.3)	98 (36.2)	179 (51.4)	58 (20.7)	0.001
Digestive System, N (%)	140 (15.6)	25 (9.2)	28 (8)	87 (31.1)	0.001
Musculoskeletal, N (%)	143 (15.9)	40 (14.8)	52 (14.9)	51 (18.2)	0.44
Circulatory System, N (%)	67 (7.5)	16 (5.9)	16 (4.6)	35 (12.5)	0.001
Congenital Diseases, N (%)	372 (41.4)	127 (46.9)	137 (39.4)	108 (38.6)	0.088
Respiratory Diseases, N (%)	121 (13.5)	13 (4.8)	9 (2.6)	99 (35.4)	0.001
Factors Influencing Health Status ^c^, N (%)	117 (13)	10 (3.7)	13 (3.7)	94 (33.6)	0.001
Other ^d^, N (%)	257 (28.6)	51 (18.8)	83 (23.9)	123 (43.9)	0.001
**Diagnoses group per Participant**, N(%)					0.001
Single diagnosis	378 (43)	119 (43.9)	169 (48.6)	90 (32.1)	
Equal or greater than 2 diagnoses	521 (58)	152 (56.1)	179 (51.4)	190 7.9)	

^a^Urban is defined by the following counties: Champaign, Cook, DeKalb, Dupage, Kane, Lake, Macon, Madison, McLean, Peoria Will, and Winnebago, according to the Illinois Primary Health Care Association (http://www.idph.state.il.us/RuralHealth/Rur_Urb_2021.pdf). The rest of the counties were considered rural. ^b^The category includes unknown, Asian, American Indian, or Alaska Native, and those who refused to provide or do not know. ^c^ Includes reasons for encounters such as dependency on supporting machines and devices, organ transplants, absence of limbs, genetics assessments, and body max index according to the International Classification of Diseases, Tenth Revision (ICD-10). ^d^ Includes conditions such as injury, poisoning, cancer, infectious or parasitic diseases, and learning/speech disabilities

Top-box results (Table 3) demonstrated families reported a positive experience with CCS, with a total average of 91% (95% CI, 94–96), which means that 6.37 of the 7 questions were answered with the top or most favorable answer. The question *“I am satisfied with how DSCC communicates with me”* shows the highest score, 93.6 ± 18.4, within this factor. A similar experience was observed for the second factor, FEP’s total average, 88% (95% CI, 93–95). Therefore, 5.28 of the 6 questions were answered with the top or most favorable selection. The highest score within this factor was *“DSCC staff support my family as we work on our person-centered plan goals*,*”* 90.5 ± 22.

**Table 3:** Descriptive statistics survey factors and questions

Factor/questions	Total^a^ items average percentage (95% CI^b^)	Average percentage ± SD^c^
**Care coordination satisfaction**^d, e^, α = 0.95	91% (94 - 96)	
DSCC staff are professional		89.5 ± 23.6
I am satisfied with how DSCC communicates with me		93.6 ± 18.4
DSCC staff help me find resources that fit my family’s needs		93.5 ± 18.6
I am satisfied with how DSCC supports my child’s education		92.4 ± 19.5
DSCC staff help me find resources for my child’s education needs		90.5 ± 21.3
DSCC staff help me address my family’s care coordination needs		90.4 ± 23
My person-centered plan includes goals that are important to our family		90.2 ± 22.4
**Family engagement and partnership**^f^ α = 0.94 DSCC staff are helpful	88% (93-95)	
		87.6 ± 22.2
I can reach my Care Coordinator when needed		90 ± 20.8
I am satisfied with DSCC’s care coordination services for my family		84.6 ± 25.2
My person-centered plan respects my family’s beliefs and preferences		89.5 ± 22
DSCC staff support my family as we work on our person-centered plan goals		90.5 ± 22
DSCC has helped improve my family’s quality of life.		87.5 ± 23

^a^Total is the average proportion of questions answered by participants with the “top” or most favorable selection (0-100 values) (McDaniel et al., 2023). For instance, the Care Coordination Satisfaction factor score of 91% shows that participants answered 6.37 of 7 questions with the most favorable or top selection. ^b^*CI* confidence interval, ^c^*SD* standard deviation. ^d^Cronbach α measures internal reliability of each factor. ^e^Items with missing values were excluded from the analysis, *N*=29 (3.2%). ^f^Items with missing values were excluded from the analysis, *N*= 139 (15.5%)

Table 4 provides correlations between factors and demographic variables. Significant correlations were < 7, implying weak correlations. Age and FEP had a positive correlation, *r* =.073, *p* <.05, while FEP and program type had a negative, *r* =-.150, *p* <.01. FEP and regions were positively correlated, *r* =.115, *p* <.01. CCS and program type were negatively correlated, *r* =.−094, *p* <.01, and positively related to regions, *r* =.126, *p* <.01. Appendix Table 7 shows individual questions correlations with demographics. The strongest correlation appears between the question “I am satisfied with DSCC’s care coordination services for my family” and program, *r* =.176, *p* <.01.

**Table 4:** Factor correlations with sample demographics

Factors	Agea	Program^b^	Gender^c^	Regions^d^	Race/Ethnicity^e^	Insurance^f^ Type	Language^g^	Diagnosis^h^ Group
Care coordination satisfaction (1)	0.028	−0.094**	0.013	0.126**	−0.018	−0.065	−0.007	0.005
Family engagement and partnership (2)	0.073*	−0.150**	0.027	0.115**	−0.008	−0.003	0.063	−0.028

^a^Continuous variable; ^b^Connect Care, Home Care and Core; ^c^Male and Female; ^d^ Urban and Rural; ^e^White, Black, Hispanic/Latino, Other (includes Asian, American Indian or Alaska Native, Unknown); ^f^Public (Medicaid) and Private (Commercial); ^g^English and Other; ^h^Single Diagnosis and ≥ 2 Diagnosis

**Care Coordination Satisfaction Items**: DSCC staff are professional; I am satisfied with how DSCC communicates with me; DSCC staff help me find resources that fit my family’s needs; I am satisfied with how DSCC supports my child’s education; DSCC staff help me find resources for my child’s education needs; DSCC staff help me address my family’s care coordination needs; My person-centered plan includes goals that are important to our family

**Family Engagement and Partnership Items**: DSCC staff are helpful; I can reach my Care Coordinator when needed; I am satisfied with DSCC’s care coordination services for my family; My person-centered plan respects my family’s beliefs and preferences; DSCC staff support my family as we work on our person-centered plan goals; DSCC has helped improve my family’s quality of life

Considering significant correlations between the factors with programs’ and regions’ variables, differences in factors’ scores were examined. Significant differences were noticed between programs for each factor, CCS, *p* <.006, and FEP, *p* <.001 (Table 5). The Connect Care program scored the highest for both factors, CCS, 93% (95% CI, 90 − 95), and FEP, 91% (95% CI, 88–93). Regions were also significantly different, with a *p* <.001 for each factor. Rural had the greatest score, CCS, 95% (95% CI, 93–97), and FEP, 91% (95% CI, 88–93).

**Table 5:** Factor scores differences by programs and regions

Variables	Care coordination satisfaction average % (95%CI^a^)	*p*- value^b^	Family engagement and partnership Average % (95%CI)	*p*- value^b^
**Programs**		0.006		0.001
Connect care	93 (90 - 95)		91 (88–93)	
Core	92 (91 - 94)		87 (84–89)	
Home care	88 (86 - 91)		82 (80–85)	
**Regions**		0.001		0.001
Urban	90 (88–91)		85 (83–87)	
Rural	95 (93–97)		91 (88–93)	

^a^*CI* confidence interval ^b^ One-Way ANOVA

## Discussion

The collaboration between the multidisciplinary internal FSC members and the FAC was pivotal to this survey’s final development and distribution. The FAC stressed the need to focus on one annual survey rather than several through a year to capture different aspects of the care coordination services provided through the three programs. This feedback proved to help impact the number of families who completed the survey. Input from families to be aware of survey fatigue was an important lesson learned.

Our 2-factor, 13-question family satisfaction survey provides a tool to measure thesatisfaction of families of CYSHCN receiving care coordination services from UIC-DSCC. The 2-factors, Care Coordination Satisfaction and Family Engagement and Partnership offer a comprehensive evaluation of families’ interactions with care coordination teams. From a quality improvement point of view, they present a better understanding of families’ satisfaction with care coordinators’ efforts, such as overall assistance to access resources according to families’ needs, including school or education resources. The 2-factors also address intervention approaches such as collaborative development and continued support of person-centered plans based on the family’s preferences, prioritization of goals, or what is important to the family and their beliefs.

Despite low score correlations between factors and demographics, significant differences were found between programs and regions across the 2 factors. Insurance variations such as public versus private among Core and Connect Care program participants and complex care needs among Home Care program participants may have contributed to these results. Further, the high scoring among those residing in a rural region suggests that families feel supported, and care coordination teams effectively interact with them and connect them with the resources in those areas. This finding aligns with Sheftall et al. (2019), who reported high satisfaction with care coordination services among caregivers of children with disabilities, particularly in areas related to facilitating access to resources.

The study had several limitations. Because of time constraints, the survey was not piloted among a smaller sample. However, the results suggest optimal validation and psychometric adequacy. Future examinations would benefit from Confirmatory Factor Analysis (CFA) to ensure the 2-factor solution. Moreover, the survey was only validated for families residing in Illinois, and other Title V-funded care coordination programs differ in program design and target population. Certainly, the two open-ended questions warrant a qualitative review. Crucial information, such as barriers to services or challenging experiences, could emerge from the narrative and inform the development of new program initiatives. The sample’s diverse representation, according to ethnicity, geographic location (rural or urban), and diagnosis, was not a surprise. However, additional exploration should consider the association of factors like race/ethnicity and diagnosis with satisfaction of care coordination services (Sheftall et al., 2019). The survey’s participants included 65 Spanish-speaking families and 30 families from other linguistic backgrounds. This linguistic diversity may have impacted items’ response rates and contributed to the absence of responses for certain items. Thus, trialing the survey in other languages should be considered in the future. Response rates may increase through this approach, and a better understanding of care coordination services and satisfaction levels from underrepresented groups (Young et al., 2019). Psychometric characteristics should be reviewed if the survey administration is expanded to other languages.

Another limitation of this study is around the measurement of quality of life. In this survey, the measurement of quality of life comes from a single question that asks families if UIC-DSCC’s care coordination services help to improve their quality of life. Response is parent reported and on a Likert scale. This is recognized to be an area that could be expanded upon further in the future.

## Conclusion

This publication summarizes the development, implementation, and validation of a 13-question survey designed to evaluate the care coordination satisfaction of families of CYSHCN enrolled in care coordination programs operated by UIC-DSCC. Family partnership is a crucial part of the care coordination process, and UIC-DSCC has learned this partnership is also critical when evaluating family satisfaction with care coordination services. The in-depth analysis of thesurvey results validated that families served by UIC-DSCC are satisfied with their care coordination services and that UIC-DSCC has helped improve their quality of life. These important measures are important when considering how to drive improvement in care of CYSHCN (Coleman et al., 2022). Based on psychometric adequacy, this instrument can advise on quality improvement actions conducted by care coordination entities working with a similar population, mainly if these care coordination programs operate outside clinical settings.

## Figures and Tables

**Fig. 1:**
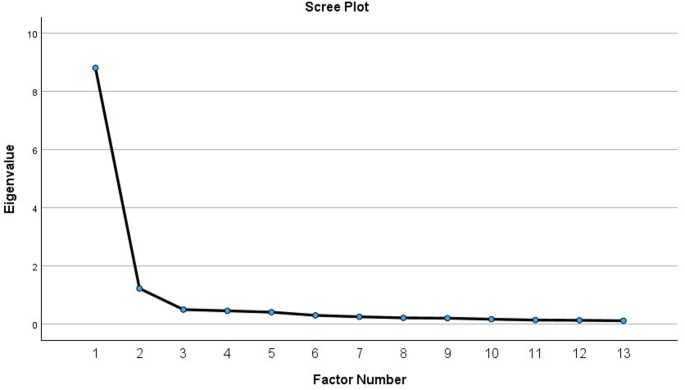
Scree plot exploratory factor analysis

## Data Availability

Not applicable.

## Appendix

See Tables 6 and 7; Fig. 1.

**Table 6:** Promax rotation 2 – factor loading

Questions	Factor 1 (Care coordination satisfaction)	Factor 2 (Family engagement and partnership)
DSCC staff are helpful		0.713
DSCC staff are professional	0.641	
I can reach my care coordinator when needed		0.814
I am satisfied with how DSCC communicates with me	0.941	
DSCC staff help me find resources that fit my family’s needs.	0.929	
I am satisfied with how DSCC supports my child’s education	0.854	
DSCC staff help me find resources for my child’s education needs	0.692	
DSCC staff help me address my family’s care coordination needs	0.663	
I am satisfied with DSCC’s care coordination services for my family		1.027
My person-centered plan respects my family’s beliefs and preferences		0.671
My person-centered plan includes goals that are important to our family	0.660	
DSCC staff support my family as we work on our person-centered plan goals		0.714
DSCC has helped improve my family’s quality of life		0.895

Excluded values < 0.40

**Table 7:** Survey items correlations with sample demographics

Factors/items Age^a^		Program^b^	Gender^c^	Regions^d^	Race/Ethnicity^e^	Insurance^f^ Type	Language^g^	Diagnosis^h^ group
*Care coordination satisfaction*								
CCS_Q2	0.029	−0.097**	0.033	0.082*	−0.024	0.006	−0.035	0.033
CCS_Q4	0.013	−0.060	0.016	0.092**	−0.011	−0.061	0.075*	0.051
CCS_Q5	0.011	−0.036	0.014	0.098**	−0.010	−0.026	−0.031	0.013
CCS_Q6	0.017	−0.074*	0.014	0.125**	−0.001	0.010	−0.065	0.001
CCS_Q7	0.023	−0.088**	−0.015	0.15**	−0.034	−0.027	−0.101**	0.004
CCS_Q8	0.032	−0.094**	0.007	0.114**	−0.017	0.020	−0.034	−0.022
PCP_Q2	0.041	−0.115**	0.014	0.137**	−0.012	0.019	−0.059	−0.045
*Family engagement and partnership*								
CCS_Q1	0.058	−0.057	0.006	0.092**	−0.034	0.052	−0.023	0.007
CCS_Q3	0.079*	−0.118**	0.008	0.113**	−0.027	0.034	−0.019	−0.034
CCS_Q9	0.096**	−0.176**	0.052	0.079*	0.019	0.109**	0.015	−0.078*
PCP_Q1	0.050	−0.150**	0.032	0.094**	0.008	0.033	0.008	−0.020
PCP_Q3	0.064	−0.128**	0.018	0.113**	−0.025	0.047	−0.042	−0.022
QOL_Q1	0.070*	0.070*	0.045	0.113**	−0.009	0.089*	0.052	−0.058

^a^ Continuous variable; ^b^Connect Care, Home Care and Core; ^c^Male and Female; ^d^ Urban and Rural; ^e^White, Black, Hispanic, Other (includes Asian, American Indian or Alaska Native, Unknown); ^f^Public (Medicaid) and Private (Commercial); ^g^English and Other; ^h^Single Diagnosis and ≥ 2 Diagnosis

CCS_Q2- DSCC staff are professional; CCS_Q4- I am satisfied with how DSCC communicates with me; CCS_Q5- DSCC staff help me find resources that fit my family’s needs; CCS_Q6- I am satisfied with how DSCC supports my child’s education; CCS_Q7- DSCC staff help me find resources for my child’s education needs; CCS_Q8- DSCC staff help me address my family’s care coordination needs; PCP_Q2- My person-centered plan includes goals that are important to our family;

CCS_Q1- DSCC staff are helpful; CCS_Q3- I can reach my Care Coordinator when needed; CCS_Q9- I am satisfied with DSCC’s care coordination services for my family; PCP_Q1- My person-centered plan respects my family’s beliefs and preferences; PCP_Q3- DSCC staff support my family as we work on our person-centered plan goals; QOL_Q1- DSCC has helped improve my family’s quality of life

**p*<.05; ***p*<.01
